# Imaging of viral neuroinvasion in the zebrafish reveals that Sindbis and chikungunya viruses favour different entry routes

**DOI:** 10.1242/dmm.029231

**Published:** 2017-07-01

**Authors:** Gabriella Passoni, Christelle Langevin, Nuno Palha, Bryan C. Mounce, Valérie Briolat, Pierre Affaticati, Elodie De Job, Jean-Stéphane Joly, Marco Vignuzzi, Maria-Carla Saleh, Philippe Herbomel, Pierre Boudinot, Jean-Pierre Levraud

**Affiliations:** 1 Virology and Molecular Immunology, INRA, Université Paris-Saclay, Domaine de Vilvert, Jouy-en-Josas F-78352, France; 2 Macrophages and Development of Immunity, Institut Pasteur, CNRS UMR 3738, 25 rue du docteur Roux, Paris F-75015, France; 3 Viral Populations and Pathogenesis Unit, Institut Pasteur, CNRS UMR 3569, Paris F-75015, France; 4 Tefor Core Facility, Paris-Saclay Institute of Neuroscience, CNRS, Université Paris-Saclay, Gif-sur-Yvette F-91190, France; 5 Viruses and RNA Interference, Institut Pasteur, CNRS UMR 3569, Paris F-75015, France

**Keywords:** Alphavirus, Chikungunya, Central nervous system, Zebrafish, Viral encephalitis, Live imaging

## Abstract

Alphaviruses, such as chikungunya virus (CHIKV) and Sindbis virus (SINV), are vector-borne pathogens that cause acute illnesses in humans and are sometimes associated with neuropathies, especially in infants and elderly patients. Little is known about their mechanism of entry into the central nervous system (CNS), even for SINV, which has been used extensively as a model for viral encephalopathies. We previously established a CHIKV infection model in the optically transparent zebrafish larva; here we describe a new SINV infection model in this host. We imaged *in vivo* the onset and progression of the infection caused by intravenous SINV inoculation. Similar to that described for CHIKV, infection in the periphery was detected early and was transient, whereas CNS infection started at later time points and was persistent or progressive. We then tested the possible mechanisms of neuroinvasion by CHIKV and SINV. Neither virus relied on macrophage-mediated transport to access the CNS. CHIKV, but not SINV, always infects endothelial cells of the brain vasculature. By contrast, axonal transport was much more efficient with SINV than CHIKV, both from the periphery to the CNS and between neural tissues. Thus, the preferred mechanisms of neuroinvasion by these two related viruses are distinct, providing a powerful imaging-friendly system to compare mechanisms and prevention methods of encephalopathies.

## Abstract (precis)

**Summary:** Imaging of neuroinvasion in zebrafish shows that chikungunya virus first infects the blood-brain barrier, whereas Sindbis virus relies on axonal transport.

## INTRODUCTION

The outcome of an infection depends crucially on the organs reached by the invasive pathogen, which entails a precarious balance between the pathogen dissemination strategy and host defenses. The central nervous system (CNS) is especially vulnerable, being highly susceptible both to virus-induced cytopathic effects and to the inflammatory response itself (Swanson and McGavern, 2015). The CNS is, however, protected by specialized barriers, notably the blood–brain barrier (BBB). Neuroinvasion is generally rare, but some viruses have evolved strategies to enter and spread within the CNS. These mechanisms have been characterized using a variety of approaches (reviewed by Koyuncu et al., 2013), and fall within three categories. Some viruses are able to infect cells of the BBB (i.e. endothelial cells that form brain microvessels) or other components of the brain–periphery interface, such as epithelial cells of choroid plexuses. This allows their release in the brain parenchyma by the infected cells and/or disruption of the barrier causing leakage of blood-borne virions. Another strategy is known as the ‘Trojan horse’ entry, whereby viruses hide in monocytes or macrophages, and exploit the ability of these cells to cross the barrier to enter the brain. Finally, various viruses travel within the numerous axons of motor or sensory neurons that connect the CNS to the periphery, using either anterograde or retrograde transport.

Persistent or ‘stable’ infection of the CNS is a rare occurrence, except for some specialized viruses. On the contrary, acute CNS infections are often caused by zoonotic pathogens, for which humans normally represent a ‘dead-end host’ (Gubler, 2001). In particular, among the arboviruses (viruses transmitted by arthropod vectors), Alphaviruses constitute a major source of viral zoonotic diseases, and often induce encephalitis. Among Alphaviruses, Eastern (EEEV) and Western Equine Encephalitis viruses (WEEV) (Paessler and Taylor, 2011) and the re-emerging chikungunya virus (CHIKV) have been associated with serious neurological manifestations (Economopoulou et al., 2009). Sindbis virus, the type species and best-studied member of the *Alphavirus* genus, has been extensively used as a viral encephalitis model in mice (Lewis et al., 1996; Lustig et al., 1988).

Despite their importance as encephalitogenic pathogens, the mechanisms of entry of Alphaviruses in the CNS are poorly understood. SINV is well known to infect neurons and has been used as a tool to trace neural circuits (Furuta et al., 2001); however, in most experimental settings it is inoculated directly in the CNS. SINV can enter the CNS from the periphery, especially in newborn mice, but this route of entry has not been the subject of detailed investigation. Experiments in mice with a luciferase reporter virus inoculated in the footpad were consistent with an entry via peripheral nerves (Cook and Griffin, 2003), but alternative entry routes were not ruled out. In the case of CHIKV, human infants infected with the virus often display signs of encephalitis (Arpino et al., 2009; Das et al., 2010). However, a murine model showed that after peripheral injection, CHIKV-infected cells were found in the meninges but not the brain parenchyma (Couderc et al., 2008). If inoculated intranasally, CHIKV could be found in neurons from the olfactory bulb (Powers and Logue, 2007), but no further spread was documented.

A major hindrance in detailed kinetic analyses of CNS viral invasion lies with the difficulty in observing these events in real time. Despite the development of virus mutants built to express reporter genes [e.g. luciferase, green fluorescent protein (GFP), mCherry], intravital imaging of virus infection in mammals is still a real challenge (Rameix-Welti et al., 2014). The zebrafish larva offers a powerful system to follow such events in a vertebrate, being small, transparent and tolerant of prolonged anaesthesia (Levraud et al., 2014). In a recent study, we performed real-time imaging of zebrafish larvae inoculated with a GFP recombinant CHIKV (Palha et al., 2013). The majority of infected cells were found in various peripheral organs; however, infection of some brain cells, which survived much longer than cells infected in the periphery, always occurred. This work demonstrated that it is possible to image neuroinvasion in zebrafish larvae from the earliest stages of the infection. In order to gain a better understanding of *Alphavirus* neuroinvasiveness, we decided to analyse the entry route of CHIKV into the CNS in zebrafish and to compare it with a SINV infection model, thus taking advantage of the extensive knowledge available for this virus. Indeed, non-replicative SINV has been successfully used to label zebrafish neurons *in vivo* (Zhu et al., 2009); moreover, we recently showed that intracranial inoculation of zebrafish larvae with a replicative SINV strain leads to extensive and progressive brain infection (Mounce et al., 2016).

Here, we first show that upon intravenous (IV) inoculation in zebrafish larvae, SINV infects multiple cell types and replicates efficiently. Moreover, in half of the infected larvae the virus reaches the CNS, where its progressive spread can be documented by *in vivo* imaging. We tested the different possible routes of invasion of the CNS for CHIKV and SINV. We looked at infection of endothelial cells of the BBB using high-resolution, whole-brain confocal imaging. Some CHIKV-infected cells were systematically found in co-localization with the brain vascular endothelium. Conversely, SINV did not infect endothelial cells or cause leakage of the BBB. We excluded macrophage-mediated entry as a significant mechanism for either virus. Macrophages were not productively infected, and their depletion did not prevent or delay CNS invasion. Time series of trunk infection suggest strongly that SINV can be transmitted from infected muscle fibres to their innervating spinal cord neurons; no such event occured with CHIKV. To test whether axonal transport favoured viral spread, we injected the viruses in the retina, which revealed efficient invasion of connected brain regions with SINV, but much less so for CHIKV.

Thus, we developed zebrafish models of infection to study viral entry in the CNS using high-resolution intravital imaging approaches. Our study reveals the conservation of neurotropism of Alphaviruses from mammals to fish based on distinct entry routes in the CNS, namely infection of endothelial cells of the BBB for CHIKV in contrast to axonal transport from peripheral nerves for SINV.

## RESULTS

### Course of SINV infection after peripheral inoculation

**Fig. 1.**
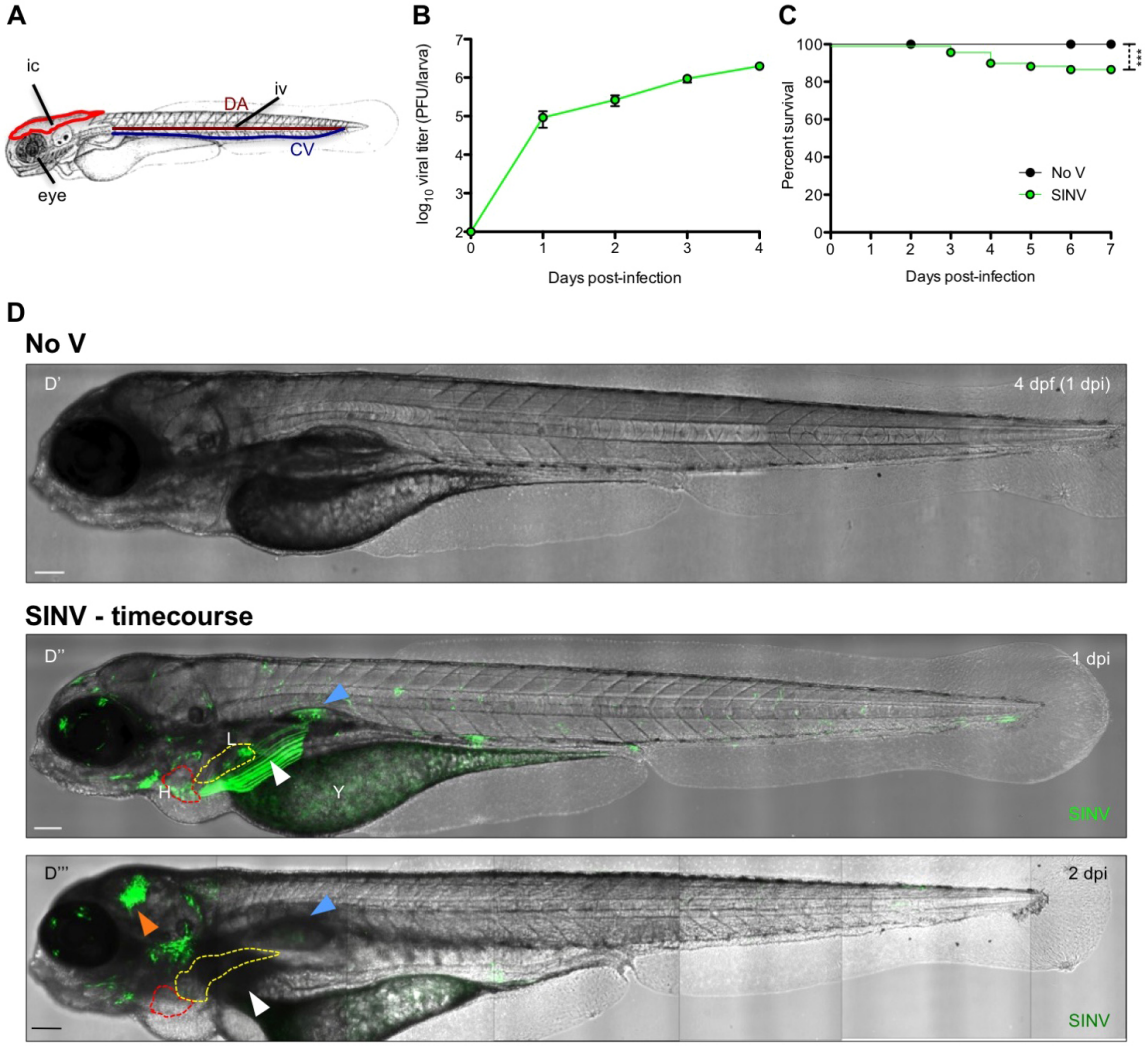
**SINV replicates in zebrafish larvae and exhibits a broad organ tropism.** (A) Scheme of a 72 hours post-fertilization (hpf) larva, showing the sites of injection: IV, intravenously in the caudal vein (CV) or the dorsal aorta (DA); IC, intracerebrally in the optic tectum; eye, in the retina. (B) Virus replication in IV-infected zebrafish larvae, assessed by titration of homogenates of whole larvae. Data represent the means±s.e.m. of five individual larvae per time point, from three experiments pooled. (C) Survival curves of control uninfected (No V) and IV-infected zebrafish larvae (SINV). Data were pooled from five independent experiments. *n*=60 larvae per group. (D) Live detection of SINV-infected cells by *in vivo* confocal imaging, with superposition of transmitted light and GFP fluorescence (maximal projection). (D′) Uninfected control (No V), 4 dpf (1 dpi). (D″,D‴) The same IV SINV-infected (SINV) larva at 1 dpi (D″) and 2 dpi (D‴). H, heart (dotted red line); L, liver (dotted yellow line); Y, yolk (note that the yolk is autofluorescent but, as shown in D′, at a nearly undetectable level using these imaging conditions; the signal in D″ corresponds to infection); white arrowhead, infection in the left pectoral muscle; blue arrowhead, infection in the swim bladder; orange arrowhead, infection in the brain. Scale bars: 50 μm. In this and all following lateral view figures, anterior is to the left and dorsal to the top.

Wild-type (WT) zebrafish larvae were inoculated IV at 3 days post-fertilization (dpf) (Fig. 1A) with ∼10^2^ plaque forming units (PFU) of SINV-GFP, similar to the protocol used for CHIKV-GFP (Palha et al., 2013). Both viruses have been engineered to induce GFP expression in infected cells under the control of an additional subgenomic promoter, reflecting expression of viral structural genes (Hahn et al., 1992; Tsetsarkin et al., 2006), and will hereafter be referred to as SINV and CHIKV, respectively.

Robust SINV replication was demonstrated by titrations from homogenates of larvae at different times post-injection (Fig. 1B). However, mortality was limited (Fig. 1C). Observation of SINV-infected larvae with a dissecting stereomicroscope showed that relatively mild and usually transient signs of disease appeared as early as 2 days post-injection (dpi), including oedema, irregular heartbeat and yolk opacification. In a minority of larvae (∼15%), these signs persisted and led to death at ∼4 dpi. The other larvae appeared to recover; however, we did not assess long-term persistence of the virus in order to avoid the additional variability entailed by feeding the larvae. Live fluorescence microscopy revealed the presence of GFP-positive (GFP^+^) cells scattered or in clusters. These infected cells were mostly observed in peripheral organs, including the liver, the heart, the yolk and in muscle fibres (Fig. 1D). Similar to what had been observed with CHIKV, infection of peripheral cells was largely transient (compare Fig. 1Dii,Diii). These results confirm that SINV replicates efficiently in zebrafish larvae and that its dispersal through the whole organism can be monitored over time via *in vivo* imaging.

### Establishment of SINV infection in the CNS

**Fig. 2.**
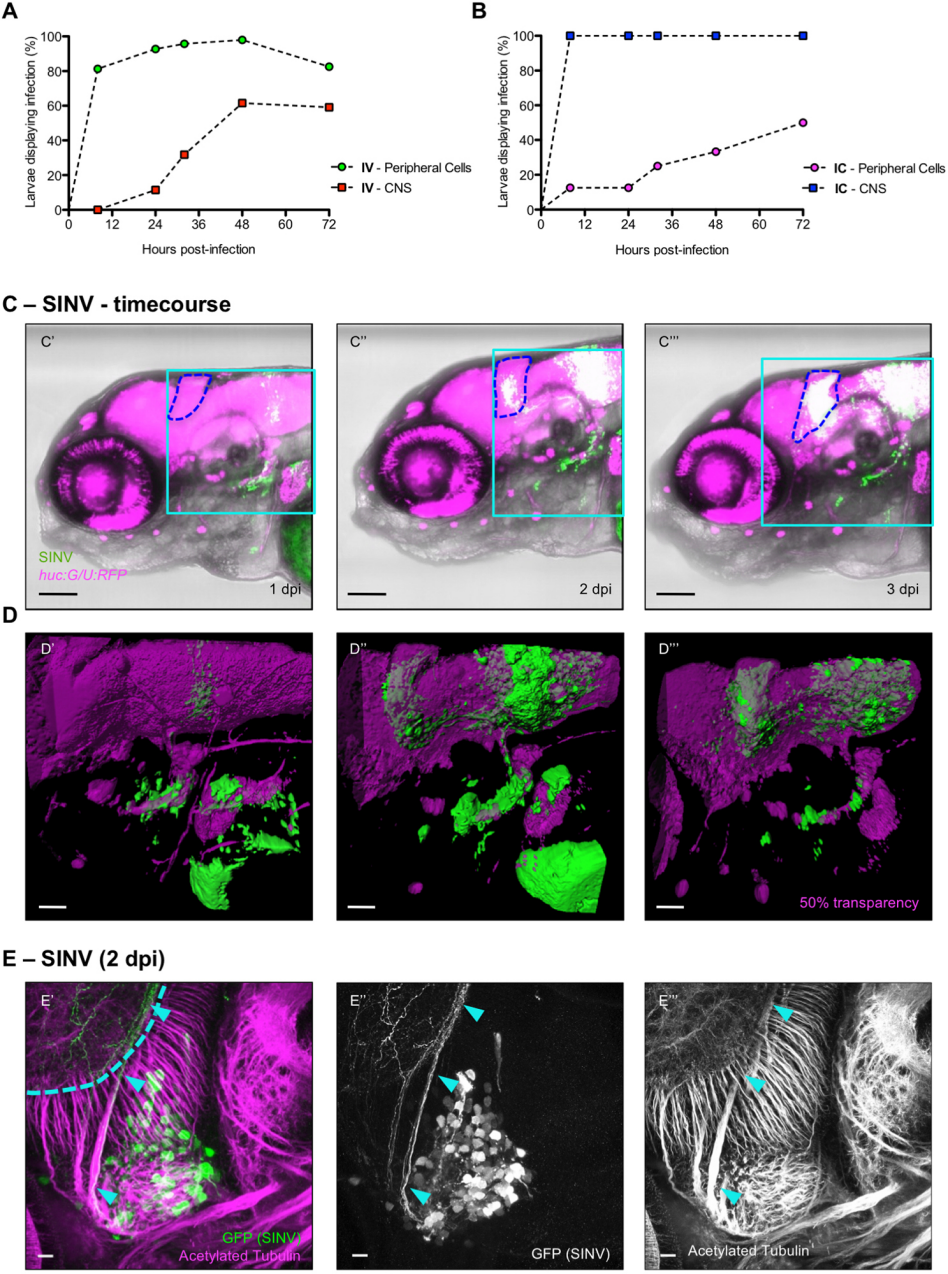
**SINV is neuroinvasive in zebrafish larvae.** (A,B) Quantification of the appearance of infected cells in the periphery (circles) and in the CNS (squares), from *in vivo* observation of *huC:G/U:RFP* larvae, following IV (A) or IC (B) inoculation. *n*=12-24 from two independent experiments pooled. (C′-C‴) Live confocal imaging of the same IV SINV-infected *huc:G/U:RFP* larva from 1 to 3 dpi, showing the progression of the infection. Superposition of transmitted light, green (infected cells) and red (neurons) fluorescence; blue dotted line, cerebellum. In this and following colour figures, red fluorescence is displayed in magenta and referred to as ‘magenta fluorescence’. The light blue squares correspond to regions shown in panels D′-D‴. Scale bars: 50 µm. (D′-D‴) 3D rendering of the brain areas shown above. Scale bars: 25 µm. (E′-E‴) Confocal image of a whole-mount immunohistochemistry processed SINV-infected WT larva, maximal projection. GFP staining (SINV-infected cells) in green; acetylated tubulin (axons) in magenta. Infected cells are in the trigeminal ganglion (TG); the axon-rich region visible behind (light blue dotted line) is the neuropile of the left optic tectum (OT). Light blue arrowheads point to the axon of an infected neuron connecting the TG to the OT. Scale bars: 10 µm.

In addition, the infection often reached the CNS (brain, spinal cord, or both). Approximately half of the larvae had obvious CNS infection by 3 dpi. To assess the kinetics and frequency of CNS infection under the fluorescence microscope reliably, we used *elavl3:Gal4/5xUAS:RFP* double-transgenic larvae (hereafter called *huC:G/U:RFP*), in which red fluorescent protein (RFP) is expressed in postmitotic neurons, thus clearly delineating CNS boundaries. The kinetics and frequency of appearance of infected cells was established by repeated observation of live infected larvae. The first infected cells to appear after IV injection, observed ∼8 h post-infection (hpi), were in the periphery (e.g. muscle cells, cells in the heart and in the liver), followed at later time points by neurons of the peripheral and the central nervous system (between 24 and 48 hpi; Fig. 2A). We could not identify any obvious pattern in these early CNS infections, which were detected in several regions of the brain (notably the telencephalon, the optic tectum, the vestibulolateralis lobe of the corpus cerebelli and the medulla) and in the spinal cord. To exclude the possibility that neurons might take longer to express GFP once infected, we performed intracranial (IC) inoculations (Fig. 1A). In that case, GFP^+^ neurons were detected as soon as 8 hpi (Fig. 2B), indicating that late appearance of CNS infection in IV-injected larvae results from the delay of virus spreading from the periphery to the CNS.

The evolution of the distribution of infected cells was evaluated by following individual larvae every 24 h with confocal imaging. Unlike foci of infection in the periphery, which generally started to decline after 2 dpi, those observed in the brain were either persistent or progressively increasing over time (Fig. 2C). Progression of infections was attributable both to growth of established foci (such as the one seen in the cerebellum in Fig. 2C′-C‴) and to spread to other neural structures (e.g. the optic tectum in Fig. 2C″,C‴). Three-dimensional (3D) rendering of the whole-brain images (Fig. 2D) strongly suggest infection of neuronal tracts connecting newly infected structures with early ones; indeed, the connection suggested by Fig. 2C,D is consistent with established wiring of the zebrafish cerebellum (Heap et al., 2013). Co-immunolabelling of SINV-positive cells and axonal projections in WT animals confirmed that neurons were infected (Fig. 2E).

For better assessment of the persistence of the infection, we followed SINV-infected larvae up to 7 dpi. Of those larvae with patent CNS infection at 3 dpi, 92% still had detectable infection by 7 dpi (11/12, excluding those that died in the interval; three independent experiments pooled).

Thus, after inoculation of SINV in the bloodstream, peripheral cells are infected first, followed by invasion of the CNS in about half the larvae. Peripheral infection is transient, whereas CNS infection is persistent and often spreads to other CNS structures, possibly along axon tracts.

Interestingly, the dynamics of CHIKV infection described previously (Palha et al., 2013) were fairly similar to those observed for SINV. However, CHIKV invasion of the CNS occurred in all larvae, without showing the same tendency to spread to other brain structures.

### CHIKV, but not SINV, infects endothelial cells of the BBB

We then proceeded to test the possible mechanism of SINV and CHIKV entry into the CNS. A number of DNA and RNA viruses have been shown to gain access to the brain through infection of endothelial cells of the BBB, including Epstein–Barr virus (Casiraghi et al., 2011), Hepatitis C virus (Fletcher et al., 2012) and West Nile virus (Xu et al., 2012). Moreover, researchers in our laboratory previously established a zebrafish infection model of a fish rhabdovirus, infectious haematopoietic necrosis virus (IHNV), and demonstrated that IHNV primarily infects endothelial cells of the vasculature. Infection of these cells in turn causes a rapid disruption of blood vessels and virus dissemination to neighbouring cells, including cells in the brain (Ludwig et al., 2011). It was therefore used as a positive control to visualize BBB disruption.

To visualize the vascular endothelium, we inoculated *fli1a:GAL4FF/5xUAS:RFP* transgenic fish (hereafter referred to as *fli:G/U:RFP*), which express RFP in all endothelial cells. Live or fixed SINV-infected larvae were imaged using confocal microscopy. Given that CHIKV is of a higher biosafety level, CHIKV-infected larvae were fixed and immunolabelled with anti-GFP and anti-RFP antibodies before confocal imaging. Likewise, for IHNV, which does not include a fluorescent reporter, larvae had to be fixed and immunolabelled using a monoclonal antibody (mAb) directed against the G protein of IHNV.

**Fig. 3.**
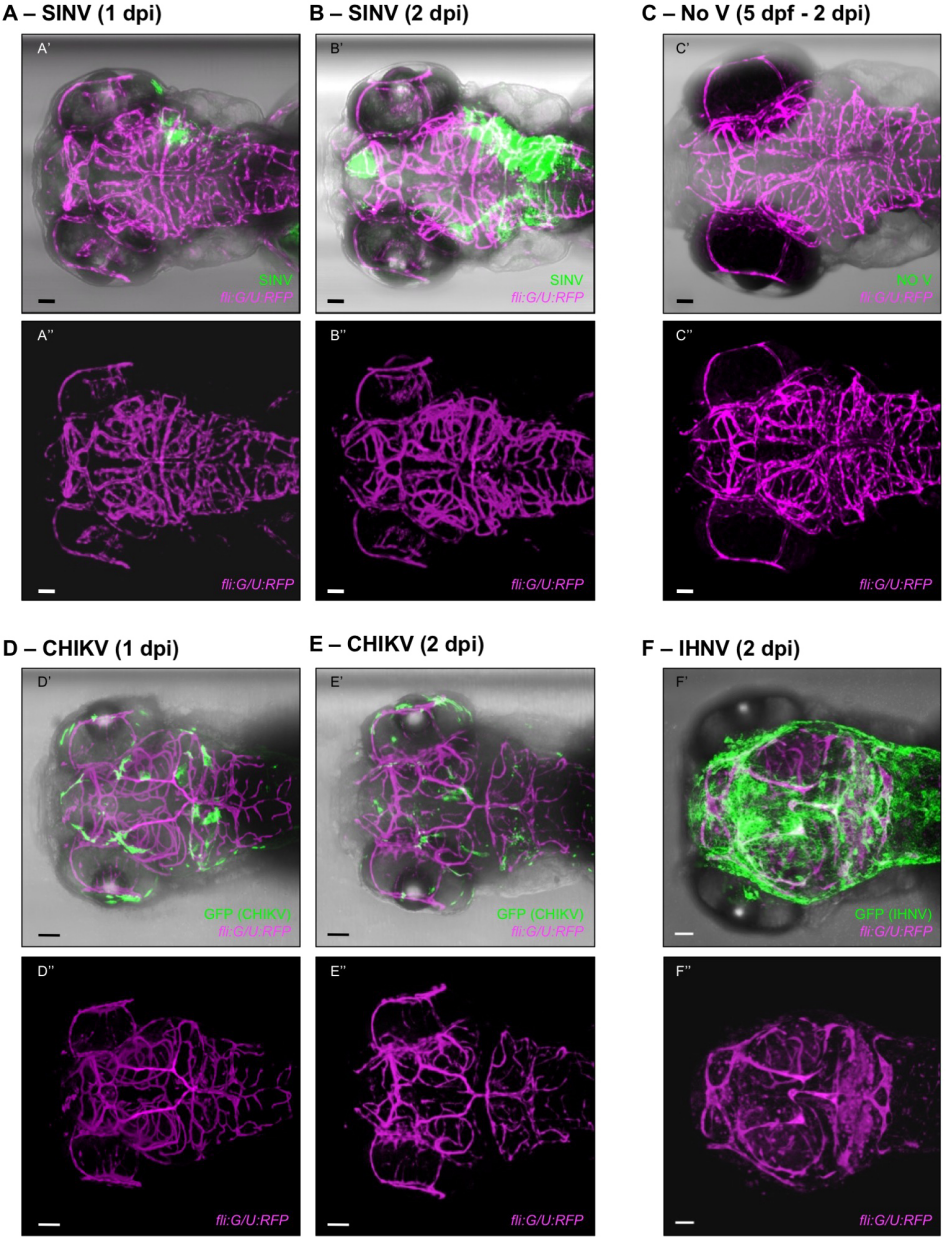
**CHIKV, but not SINV, infects the brain microvascular endothelium.** Dorsal views of *fli:G/U:RFP* larvae, with infected cells in green and endothelial cells shown in magenta; confocal imaging, maximal projections. For all panels, the top image (with ′) shows superposition of transmitted light with green and magenta fluorescence, and the bottom image (with ″) shows only the magenta fluorescence to provide better visualization of the vasculature. Scale bars: 50 μm. (A,B) Live imaging of the same SINV-infected larva at 1 dpi (A) and 2 dpi (B). See also Fig. S1A, Movies 1 and 2, for a view through the *z*-stack. (C) Live imaging of control uninfected (No V) larva. (D,E) Fixed CHIKV-infected larvae at 1 dpi (D) and 2 dpi (E). See also Fig. S1B and Movie 4, for a view through the *z*-stack. (F) Fixed IHNV-infected larva at 2 dpi. See also Movie 5, for a view through the *z*-stack. In this and all following dorsal view figures, anterior is to the left.

Live imaging of SINV-infected larvae did not reveal any infection of endothelial cells of the BBB (Fig. 3A,B). Sometimes, intense infection of the parenchyma at 2 dpi or later made it difficult to ascertain that adjacent RFP-positive (RFP^+^) cells did not express GFP (Fig. 3B′; Movie 1); however, in that case, imaging of the same larvae at an earlier stage of infection (1 dpi) clearly showed that at this crucial time, no endothelial cell was infected (Fig. 3A′; Fig. S1A; Movie 2). The vascular network was not perturbed in SINV-infected fish (compare Fig. 3B″,C″), as was verified up to 7 dpi (not shown). The same conclusions were reached by imaging fixed SINV-infected larvae. To ensure optimal imaging of deep brain vessels, some larvae were processed using the recently developed CLARITY protocol (Chung et al., 2013; Yang et al., 2014) and then entirely imaged at high resolution. Again, imaging revealed no apparent disruption of brain microvasculature in SINV-infected animals. Infected cells were sometimes adjacent to cells of the vasculature, especially in the periphery, but no co-localization was observed (for 15 animals examined in the brain, in three independent experiments, and for 13 animals examined in the periphery; Fig. S1C′-C‴; Movie 3).

By contrast, infected endothelial cells were detected in the brain vasculature of every CHIKV-infected larva at 1 or 2 dpi (Fig. 3D,E; Fig. S1B; Movie 4). The vascular network looked intact, consistent with our previous measurement of the kinetics of death of infected cells (Palha et al., 2013), which usually occurred between 2 and 3 dpi for endothelial cells in the periphery.

Finally, with IHNV, all stained fish showed many disrupted vessels (Fig. 3F; Movie 5), confirming that an abnormal vascular pattern is readily observable with these imaging conditions.

**Fig. 4.**
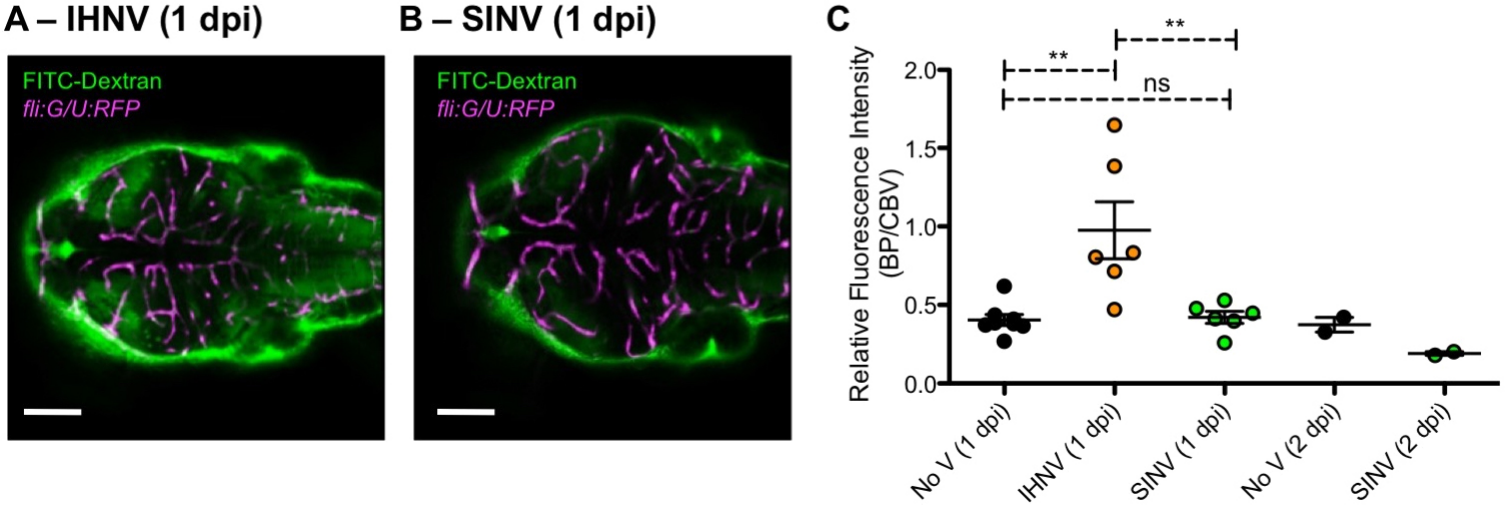
**SINV does not induce BBB leakage.** *In vivo* assay for BBB permeability. Uninfected controls, IHNV- and SINV-infected *fliG/U:RFP* larvae were injected IV at 1 and 2 dpi with 10 kDa FITC-dextran. One hour later, live confocal imaging of the brain was performed, to compare FITC intensity within the CBV and BP. (Injections at 2 dpi were performed only on uninfected controls and SINV-infected larvae, because of early death of IHNV-infected larvae.) (A,B) Representative images of IHNV-infected (A) and SINV-infected (B) larvae; single confocal plane at same depth. Scale bars: 100 μm. (C) Ratios of FITC fluorescence intensities measured in BP and CBV. *n*=2-5 larvae per group; two separate focal planes per larva.

To rule out the possibility that SINV infection could loosen the BBB without direct endothelial cell infection, and thus allow leakage of virus to the brain, we performed a dye exclusion experiment on IHNV- and SINV-infected larvae. Again, because CHIKV is a Biosafety Level 3 pathogen, these experiments requiring *in vivo* imaging were not performed on CHIKV-infected larvae. We injected fluorescein isothiocyanate (FITC)-dextran (10 kDa) IV at 1 and 2 dpi and measured the relative fluorescence intensity (FI) between the brain parenchyma (BP) and the cerebral blood vasculature (CBV). Our measurements showed a significant increase in FI in the BP of IHNV-infected larvae but not in that of SINV-infected larvae (Fig. 4). Thus, the tightness of the BBB is not detectably compromised in SINV-infected larvae.

In conclusion, our data show that SINV does not infect or disrupt the BBB endothelium to gain access to the CNS. CHIKV, on the contrary, systematically infects endothelial cells of the vasculature, thus gaining an access route to the nearby parenchyma.

### Macrophages play no role in entry of SINV or CHIKV into the CNS

A second possible mechanism by which viruses can enter the CNS is the so-called ‘Trojan horse’ strategy, where viruses infect macrophages, monocytes, or both, and exploit their natural ability to cross the BBB. To test this possibility, we used *mpeg1:GAL4FF/UAS-E1b:Eco.NfsB-mCherry* transgenic larvae (hereafter called *mpeg:G/U:Nfsb-mCherry*). In this transgenic line, macrophages (including microglia) express a cytosolic red fluorescent protein fused to a bacterial nitroreductase, thus allowing both fluorescence imaging of macrophages and their depletion upon addition of metronidazole to the water, as previously described (Davison et al., 2007).

**Fig. 5.**
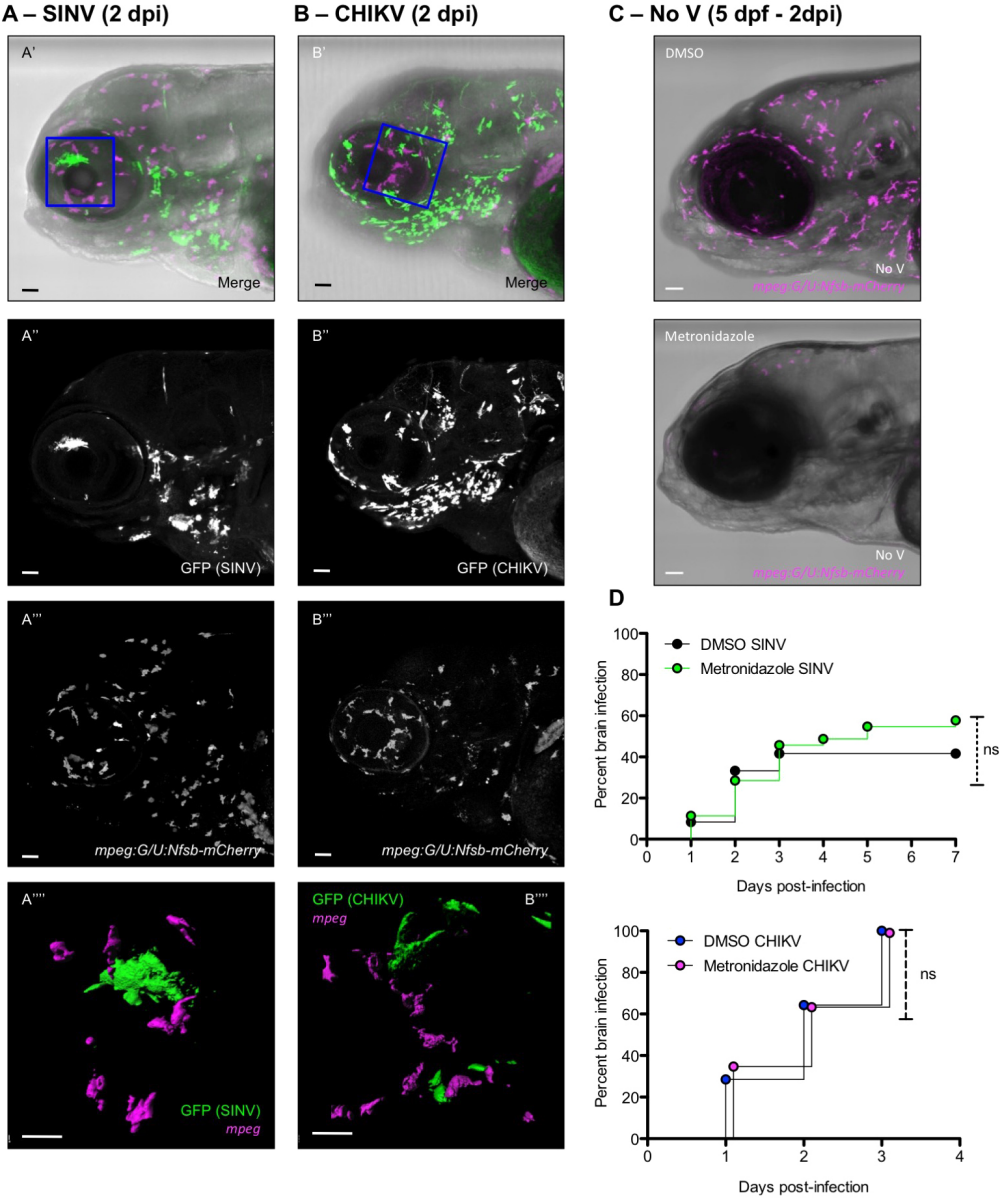
**Macrophages are not infected by SINV or CHIKV and not required for neuroinvasion.** (A,B) Whole-mount immunohistochemistry of SINV-infected (A) or CHIKV-infected (B) *mpeg:G/U:Nfsb-mCherry* (macrophages in magenta) larva at 2 dpi. Lateral views of the head; confocal imaging, maximal projection. (A′,B′) Merge of transmitted light with green (infected cells) and magenta (macrophages) fluorescence. (A″,B″) Green fluorescence showing infected cells. (A‴,B‴) Magenta fluorescence showing macrophage distribution. (A‴′,B‴′) Magnification of the area boxed in A′ and B′; green and magenta fluorescence, 3D rendering slightly tilted. Scale bars: 50 µm (A′-A‴,B′-B‴), 25 µm (A″″,B″″). (C) Macrophage depletion in *mpeg:G/U:Nfsb-mCherry* larvae. Superposition of transmitted light and magenta (macrophage) fluorescence, maximal projection, lateral view of the head, in a DMSO-treated control (top) or 2 days after treatment with metronidazole (bottom). (D) Impact of macrophage depletion on occurrence of neuroinvasion. *mpeg:G/U:Nfsb-mCherry* larvae were treated with DMSO or metronidazole, before injection with SINV (top graph) or CHIKV (bottom graph). Percentage of brain-infected larvae. *n*=36 from three independent experiments pooled. ns, not significant (Log-rank test).

Confocal microscopy after SINV or CHIKV infection of *mpeg:G/U:Nfsb-mCherry* larvae, without macrophage depletion, revealed no cell with co-localization of green and red fluorescence (for 11 SINV-infected animals examined, in four independent experiments, and for seven CHIKV-infected animals examined, in two independent experiments; Fig. 5A,B); thus macrophages were not productively infected by either virus. They displayed their characteristic contorted shape and showed no evidence of apoptosis.

We then depleted macrophages before infection by addition of metronidazole from 2 to 3 dpf. This results in a deep macrophage depletion that lasts in the periphery and CNS for at least 2 days (Fig. 5C). A progressive recovery then occurs, first observed in the caudal haematopoietic tissue, as we documented previously (see Fig. S5D of Palha et al., 2013). Macrophage-depleted SINV-infected larvae displayed a slight increase in disease severity (disease score 5.2±1.1 compared with 3.6±1.0 in controls; mean±s.e.m.; *n*=24 per group; see Materials and Methods) but no significant increase in mortality (data not shown), in a very similar fashion to what had been observed with CHIKV (Palha et al., 2013). When CNS infection was specifically assessed, it clearly occurred in macrophage-depleted larvae, both with SINV and CHIKV. By 7 dpi, both controls and treated fish displayed a similar rate of infection (∼50%) in the CNS upon SINV infection. In the case of CHIKV, already at 3 dpi, 100% of larvae displayed CNS infection in both controls and treated fish (Fig. 5D).

We could therefore exclude the possibility that macrophages play a significant role in the entry of either SINV or CHIKV into the CNS.

### Evidence for efficient axonal transport of SINV

**Fig. 6.**
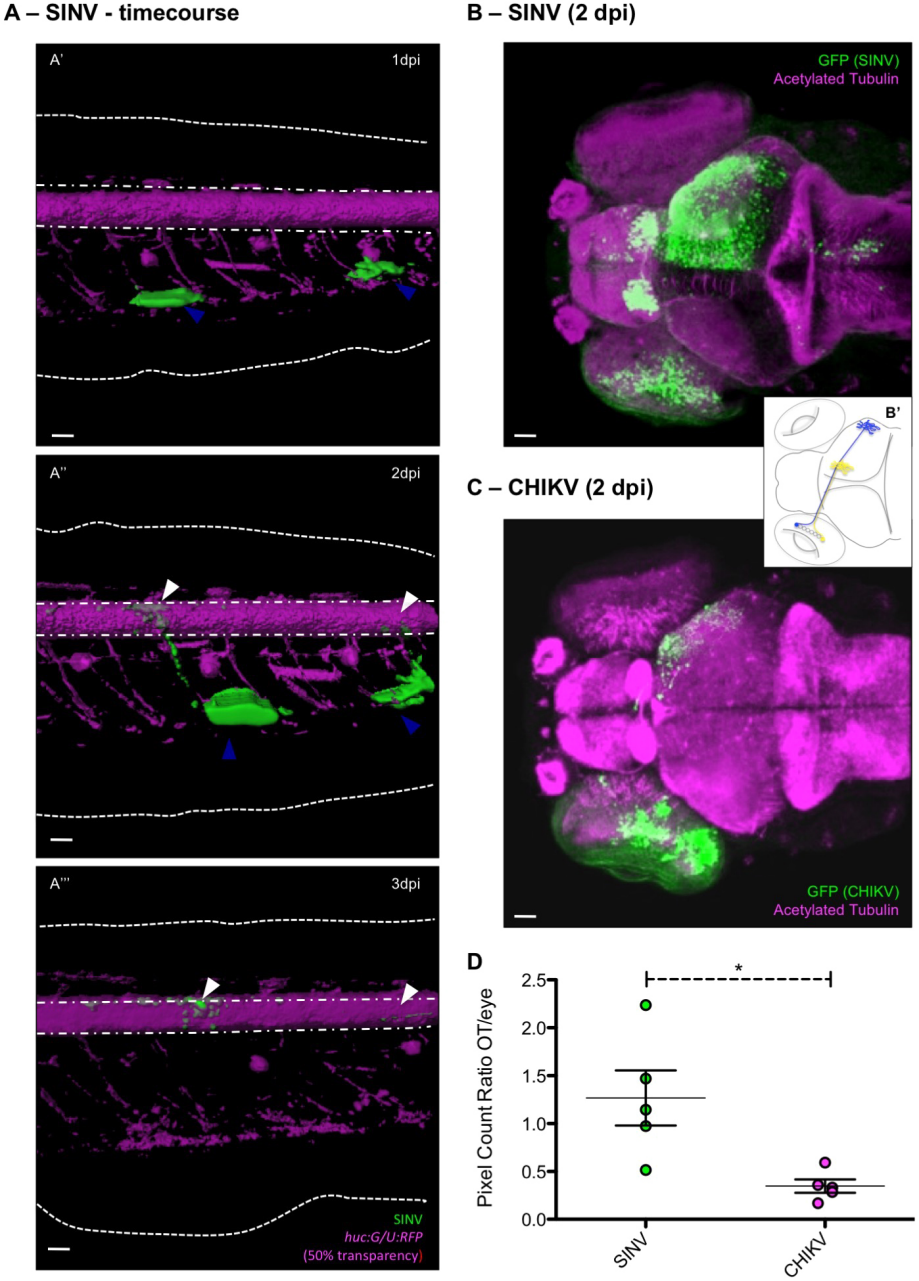
**Efficient axonal transport of SINV.** (A) Infection of muscle cells and connected spinal cord neurons in the tail region of a *huc:G/U:RFP* larva. Live confocal imaging, maximal projection, 3D rendering, with superposition of green (infected cells) and magenta (neurons) fluorescence. Same larva imaged at 1 (A′), 2 (A″) and 3 dpi (A‴). Dotted white lines indicate the limits of the fins; dash-dotted lines the limits of the spinal cord. Scale bars: 50 µm. (B,C) Assay for axonal transport to the contralateral optic tectum after inoculation of SINV (B) or CHIKV (C) in the left retina. Confocal imaging of fixed infected larva at 2 dpi, with superposition of green (infected cells) and magenta (acetylated tubulin) fluorescence; maximal projections. Scale bars: 50 μm. (B′) Scheme of the projection of the retinal neurons to the optic tectum. (D) Ratio of GFP fluorescence intensities measured in the eye and the contralateral optic tectum, after SINV or CHIKV inoculation. *n*=5 from two independent experiments pooled.

Several viruses infect and replicate in peripheral nerves (Koyuncu et al., 2013), which are connected to neurons of the CNS and act as a springboard for efficient entry and replication in the brain. In the case of SINV infection, we sometimes observed infection of peripheral neurons, such as trigeminal ganglion (TG) cells (Fig. 2E). However, because of the vast area innervated by the TG and other head ganglions, and the complexity of their connections to the brain, it was difficult to identify the sequence of events. By contrast, the spinal cord invasion offered a more tractable system. Early infection of muscle cells followed by appearance of GFP^+^ cells among motor or sensory neurons at the corresponding level of the spinal cord (Fig. 6A) was a common occurrence (observed in 44 out of 57 larvae with muscle infection, examined twice a day under the fluorescence stereomicroscope; pooled data from four independent experiments). These observations strongly suggest axonal spreading of SINV from the periphery to the CNS. By contrast, although infection of muscle fibres occurs occasionally with CHIKV, no infection of the spinal cord was observed with this virus, unless specifically injected there (not shown).

To investigate axonal transport, we injected SINV or CHIKV into the retina, between the ganglion cell layer and the outer nuclear layer (Fig. 1A), to take advantage of the well-known connectivity of the visual system (Grove, 2008; see scheme, Fig. 6B′). Fish injected in the eye did not show an increase in mortality, and overall disease scores were comparable to those of IV-inoculated fish (data not shown). We could not prevent some leakage of the inoculum into other tissues, which resulted in infection of areas close to the injected eye. Axonal transport resulted instead in infection of the contralateral optic tectum (Fig. 6B). All SINV-infected larvae (*n*=36) displayed GFP in the retina at 1 dpi, leading to infection of the contralateral optic tectum for 45% of the injected fish. In addition, spreading of infection to other brain structures, such as the habenula in Fig. 6B, were often observed. By contrast, ∼40% of CHIKV eye-injected larvae showed contralateral optic tectum infection, but of a lesser magnitude than for SINV infections (Fig. 6C). In this case, the foci of infection remained small, with no obvious spread to other CNS areas; quantification of infected areas showed a roughly tenfold decrease in efficiency of optic tectum invasion by CHIKV compared with SINV (Fig. 6D). Thus, SINV propagates efficiently, and CHIKV much less so, via axonal transport upon intraocular injection.

### Mechanisms of entry of other virus strains

We tested the generality of our findings using a different strain of each virus.

The clinical CHIKV-115 strain, which bears no fluorescent reporter gene, harbours four differences in protein sequence compared with CHIKV-GFP, including the A226V mutation in E1, shown to affect vector specificity (Tsetsarkin et al., 2007). The two viruses were known to replicate in a similar manner in zebrafish (Palha et al., 2013). The SINV339-mCherry strain, encoding a red fluorescent reporter, has eight amino acid differences compared with SINV-GFP, including the H55Q change in E2, linked to attenuation in mice (Lustig et al., 1988). It was more virulent in zebrafish than the SINV-GFP strain (Fig. S2A,B) and was therefore inoculated at a lower dose (∼10 PFU) for comparison of tropism.

We infected *fli1a*:eGFP transgenic larvae, which express GFP in endothelial cells, with either SINV339-mCherry or CHIKV-115. We fixed the larvae at 1 and 2 dpi, and imaged the entire brain by confocal microscopy after immunolabelling. SINV339-mCherry (unlike SINV) reached the CNS in 100% of infected larvae; however, no co-localization was observed between infected cells and endothelial cells (Fig. S2C). CHIKV-115, detected with an anti-capsid antibody, was systematically observed in some brain microvessel endothelial cells, despite the lower signal given by the anti-capsid antibody used (Fig. S2D).

Altogether, the analysis of the possible CNS entry routes indicates infection of the BBB endothelium as the main route of entry of CHIKV, whereas SINV would preferentially enter via infection of peripheral nerve termini and axonal transport.

## DISCUSSION

Invasion of the CNS is a key event during many viral infections and often a matter of life or death for the host. It is, however, difficult to follow this phenomenon in mammals, hindering the design of preventive strategies.

In the present work, we used zebrafish to characterize the infection course of SINV, a well-established model for the study of viral encephalitis. We focused on its neuroinvasion and compared it with that of CHIKV. This was studied following IV injections, which mimic the natural entry route of these mosquito-transmitted viruses, even though it results in more inter-individual variability than direct IC inoculation. We found that, upon IV inoculation of SINV, infected cells appeared in the periphery as early as 8 hpi, always before infected neurons of the CNS. Infection spontaneously declined in the periphery, probably as a result of the host response. Indeed, qRT-PCR analysis of whole larvae revealed strong induction of type I interferon (IFN) and IFN-stimulated genes of an amplitude and kinetics comparable to those elicited by CHIKV (not shown; a detailed analysis of the response will be reported in a separate publication). By contrast, infection persisted in the CNS. Overall, the disease signs and kinetics of SINV infection in zebrafish were similar to those previously observed in CHIKV-infected fish (Palha et al., 2013), suggesting that many of the mechanisms that determine disease onset might be conserved between related Alphaviruses. However, the entry and spread in the CNS differed. CNS entry of CHIKV was more frequent than for SINV (100 *vs* 50%), but then SINV infection of the CNS was more active, increasing in size and advancing to other brain areas. This is consistent with the different routes of CNS entry suggested by our study: via infection of endothelial cells of the BBB for CHIKV, and via infection of peripheral nerves and peripheral transport for SINV.

What routes of access to the CNS could have been used by these viruses apart from the three we tested? Direct access via an immature, leaky BBB can be dismissed. Fleming et al. (2013) characterized the progressive maturation of the BBB in zebrafish. At 3 dpf, the time at which larvae were injected, medium molecular weight molecules (∼900 Da) are already blocked from entry into the CNS (Fleming et al., 2013), thus excluding passive transport of the much bigger (52 MDa) viral particles from the blood. In some cases, viruses have been shown to enter via the choroid plexus (ChP) (e.g. HIV; Falangola et al., 1995), the region of the brain where the cerebrospinal fluid is formed. Studies on morphogenesis have shown that in zebrafish the ChP develops at the dorsal midline on the fourth ventricle and shifts towards the ear level at ∼3 dpf (García-Lecea et al., 2008). Here we found that commonly infected areas in the brain were rather lateral or distant from the ChP (e.g. olfactory bulb, optic tectum, medulla), which strongly suggests that neither SINV nor CHIKV enters the brain via prior infection of the forming ChP.

In the case of SINV, the vasculature was shown to remain intact. Moreover, infection of endothelial cells of the brain microvessels was never observed. Injection of a dye confirmed the integrity of the BBB, as leakage into the brain parenchyma did not occur in SINV-infected larvae. IC injections revealed little or no infection of cells outside the CNS, thereby corroborating the observation that SINV does not compromise the integrity of the BBB. CHIKV was, on the contrary, capable of infecting endothelial cells of both the periphery and the brain microvasculature, thus gaining an easy access to brain parenchyma. Biosafety-imposed limitations to live imaging of CHIKV infection have so far precluded direct imaging of the progression of CHIKV infection from the vasculature to the adjacent brain parenchyma, but that is an objective we will be pursuing.

Importantly, when we tested different strains of either SINV or CHIKV, the key property of infection of brain endothelial cells was conserved. Unexpectedly, the SINV339-mCherry strain was found to be significantly more virulent than its GFP counterpart. This was unexpected because the TE12 strain, on which the GFP virus is based, possesses the envelope genes from the more virulent NSV strain (Lustig et al., 1988). However, as NSV had been derived from AR339 by IC passages in mice, these virulence determinants might very well be mouse specific. Alternatively, this could also be attributable to the different position of the fluorescent reporter gene, which also has an influence on virulence (Sun et al., 2014). This is an issue that we are currently investigating. Nevertheless, we showed that irrespective of the strain used, SINV and CHIKV differ in their CNS cell tropism, which in turn determines their different mechanisms of entry into the CNS.

We also showed that neither SINV nor CHIKV targets macrophages. Macrophage depletion assays induced a slightly higher disease severity in treated fish, as in the CHIKV model, but did not prevent the virus from accessing the CNS. These results show that, even though macrophages might contribute in part to the control of SINV infection, they are not required for the virus to reach the CNS.

The observations we collected from SINV-inoculated larvae therefore strongly support the hypothesis that viral entry into the CNS occurs after infection of peripheral nerves. We observed, in some cases, infection of TG neurons that innervate the head and are connected to the brain, or infection of muscle cells followed by infection of corresponding motoneurons. These results are in accordance with the aforementioned study of mice infected with a luciferase-recombinant SINV strain, where viral replication in the nose or the spinal cord was shown always to precede infection in the brain (Cook and Griffin, 2003). Our eye inoculation experiments also showed that SINV, much more than CHIKV, can be transported efficiently by axons; a feature that is also consistent with the progressive spread to several brain substructures commonly observed with SINV infection. Our next challenge will be to achieve *in vivo* imaging of virions and of axonal transport of the virus, which will require prolonged development of new tools.

Understanding the entry mechanism of a certain pathogen into the CNS has always been hindered by the difficulties in visualizing the progression of the infection. Moreover, high numbers of animals and organ/tissue samples are generally needed at different time points to avoid missing important cues. The combined use of recombinant virus strains with fluorescent-reporter zebrafish lines, on the contrary, allowed us to gain single-cell resolution details at the whole organism level and with minimal invasiveness for the host over the full course of infection. The characterization of two viruses that rely on different entry routes to the CNS, along with future study of other neurotropic viruses in zebrafish, provides a powerful tool to test new antiviral therapeutics *in vivo*.

## MATERIALS AND METHODS

### Ethical statement

Animal experiments were conducted according to European Union guidelines for handling of laboratory animals (http://ec.europa.eu/environment/chemicals/lab_animals/home_en.htm). All protocols were approved by the Ethical Committee for Animal Experimentation of Institut Pasteur – CEEA 89 and the French Ministry of Research and Education (permit #01265.03). During injections or live imaging sessions, animals were anaesthetized with tricaine (Sigma-Aldrich, A-5040); at the end of the experimental procedures, they were euthanized by anaesthetic overdose.

### Fish lines and husbandry

Zebrafish embryos were raised according to standard procedures as previously described (Westerfield, 2000; Levraud et al., 2008). WT AB zebrafish were initially obtained from ZIRC (Eugene, OR, USA). The following transgenic and mutant lines were also used: Tg*(elavl3:Gal4)^zf349^* (Akerboom et al., 2012), Tg*(fli1a:Gal4FF)^ubs4^* (Zygmunt et al., 2011), Tg*(5xUAS:RFP)^nkuasrfp1a^* (Asakawa et al., 2008), Tg*(mpeg:Gal4FF)^gl25^* (Ellett et al., 2011), Tg*(UAS-E1b:Eco.NfsB-mCherry)^c264^* (Davison et al., 2007) and Tg(*fli1a*:eGFP)^y1^ (Lawson and Weinstein, 2002). Owing to silencing issues of some UAS-driven transgenes, breeders were carefully screened to select those whose progeny yielded full expression; correct fluorescence expression by larvae was checked before experiments.

Eggs obtained by natural spawning were bleached and raised at 28°C in Volvic source water. Eggs were raised in 1-phenyl-2-thiourea (PTU)/Volvic (Sigma-Aldrich; 0.003% final) from 24 hpf onwards to prevent melanin pigment formation. In control experiments, we verified that overall viral replication (measured by qRT-PCR) and the frequency of brain infection remain unchanged in the absence or presence of PTU. At 3 dpf, immediately before infections, larvae that had not hatched spontaneously were manually dechorionated.

### Viruses

SINV and CHIKV viruses were produced on BHK cells [originally obtained from American Type Culture Collection (ATCC), #CC-L10], according to Hardwick and Levine (2000). The SINV-GFP backbone is from the hybrid TE12 strain, with non-structural and capsid regions from the laboratory-adapted Toto1101 strain and most of the envelope region from the NSV strain isolated after six intracerebral passages of AR339 in mice (Lustig et al., 1988). It harbours a 3′ genomic insertion of the eGFP gene under the control of a second subgenomic promoter (Hahn et al., 1992). The SINV339-mCherry backbone comes from the low-passage AR339 strain, with a self-cleavable mCherry inserted between the capsid and pE2 regions (Sun et al., 2014). CHIKV-GFP corresponds to the CHIKV-LR 5′GFP virus of Tsetsarkin et al. (2006). It contains an insertion of the GFP-encoding sequence between the two main open reading frames of CHIKV under the control of an additional subgenomic promoter. The LR backbone used (#EU224268) derives from the OPY1 strain, a 2006 clinical isolate from La Réunion. CHIKV-115 is another clinical isolate from La Réunion (Schuffenecker et al., 2006) (#AM258990). Heat-adapted IHNV strain 25.70 was produced on EPC cells (ATCC #CRL-2872) as described previously (Ludwig et al., 2011).

### Injections and disease scores

Injections and handling of larvae were performed as described by Levraud et al. (2008). Briefly, zebrafish larvae aged 70-72 hpf were inoculated by microinjection of ∼10^2^ PFU viral SINV or CHIKV particles (∼10 PFU in the case of SINV339-mCherry) (∼1 nl of supernatant from infected BHK cells, diluted with PBS to 10^8^ PFU/ml). Injections were performed in the caudal vein or aorta (IV) or in the left optic tectum (IC) or in the left retina (eye) (Fig. 1A). Larvae were then distributed in individual wells of 24-well culture plates with 1 ml water containing PTU, kept at 28°C and inspected at least daily with a stereomicroscope until 7 dpi. Clinical signs of infection were assessed first on aware animals, which were then anaesthetized for better observation. Quantitative assessment of the clinical status was based on a precise list of criteria, as previously described (Palha et al., 2013). Briefly, clinical signs were assessed blindly, yielding a disease score ranging from 0 (no disease sign) to 15 (dead or terminally ill). The signs evaluated included the following: ability to maintain equilibrium (on 1 point), response to touch, body shape, blood flow, cardiac rhythm, presence of oedema, inflation of the swim bladder, and opacity of the yolk (each on 2 points). For ethical reasons, all larvae used in the experiments were euthanized by anaesthetic overdose at 7 dpi.

IHNV infections were performed as described by Ludwig et al. (2011). Briefly, larvae were injected IV with 10^2^ PFU of IHNV25.70, distributed in individual 24-well plates and incubated at 24°C.

Injections of FITC-dextran (10 kDa; Sigma) were performed at 2 dpi on control uninfected larvae, IHNV- and SINV-infected larvae. Larvae were imaged 1 h post-injection (see below).

### Viral titrations

SINV-infected larvae were anaesthetized and homogenized at 2 dpi. Dilutions of homogenate supernatant were prepared in serum-free Dulbecco's modified Eagle's medium (DMEM) and used to inoculate confluent monolayers of Vero-E6 cells (ATCC #CRL-1586) for between 30 min and 1 h at 37°C. Cells were then overlaid with 0.8% agarose in DMEM containing 1.6% newborn calf serum. Samples were incubated for 48 h. Following incubation, cells were fixed with 4% formalin and revealed with Crystal Violet solution [10% Crystal Violet (Sigma), 20% ethanol]. Plaques were enumerated and used to back-calculate the number of PFU per larva.

### *In vivo* confocal imaging

For *in vivo* imaging, 5-10 larvae were anaesthetized with 112 µg/ml tricaine and immobilized in ∼1% low-melting-point agarose in the centre of a 35 mm glass-bottomed Ibidi dish, then covered with ∼2 ml water containing tricaine. Transmitted light/fluorescence imaging was performed using a Leica SPE inverted confocal microscope using a 10× ACS APO dry objective (NA 0.30). Imaging was typically performed at 26°C, with 2 μm step *z*-stacks. Fish were imaged every day beginning at 1 dpi up to 4-5 dpi, with imaging sessions typically lasting 10-15 min; control uninfected larvae were always included.

### Whole-mount immunohistochemistry and imaging of fixed samples

Zebrafish larvae from 4 to 7 dpf were fixed in freshly prepared formaldehyde 4% (wt/vol; in PBS) overnight at 4°C. Fixed samples were washed twice in PBS containing 0.1% Tween 20 (PBSt), and whole-mount immunohistochemistry was performed as described by Svoboda et al. (2001). The following primary antibodies were used: chicken polyclonal to GFP (Abcam, ab13970, 1:500); mouse mAb to acetylated tubulin (Sigma, T7451, 1:1000); rabbit polyclonal to DsRed (Clontech, 632496, 1:500), which also labels the mCherry protein; 19B7 mouse mAb antibody specific against IHNV G protein (1:500) (Biacchesi et al., 2002); and a mouse mAb to Alphavirus capsid (1:200) (Greiser-Wilke et al., 1989). Secondary antibodies used were as follows: Alexa 488-labelled goat anti-chicken (Invitrogen, A11039, 1:500); Alexa Cy3-labelled goat anti-mouse (Jackson Immunoresearch, 115-166-003, 1:500); Alexa Cy5-labelled goat anti-mouse (Jackson Immunoresearch, 115-176-072, 1:500); and Cy3-labelled goat anti-rabbit IgG (Jackson Immunoresearch, 115-166-003, 1:500). Nuclei were stained for 45 min at room temperature with DAPI in PBSt (Sigma; 5 mg/ml). Fixed embryos were progressively transferred into 80% glycerol before imaging. Images were acquired with the Leica SPE inverted confocal microscope, using a 10× dry ACS APO objective (NA 0.30) or a 40× ACS APO oil immersion objective (NA1.15) for subregions (e.g. Fig. 2E), and *z*-stacks of a maximum of 150 μm in 2 μm steps were obtained. Image processing (maximal projections and reconstruction of whole embryos) was carried out with Adobe Photoshop CS6 software. 3D rendering of confocal stacks was done using BitPlane Imaris software, using default parameters except that red colour transparency was set at 50%.

### Passive clarification (CLARITY)

Zebrafish larvae at 4-7 dpf were fixed and washed as previously described. Samples were then infused in a precooled (4°C) solution of freshly prepared hydrogel monomers [0.01 M PBS, 0.25% VA-044 initiator (wt/vol), 5% dimethyl sulphoxide (DMSO; vol/vol), 1% paraformaldehyde (wt/vol), 4% acrylamide (wt/vol) and 0.0025% *bis*-acrylamide (wt/vol)] for 2 days at 4°C. After degassing the samples, the hydrogel polymerization was triggered by replacing atmospheric oxygen with nitrogen in a desiccation chamber for 3 h at 37°C. Samples were cleaned from superfluous hydrogel and transferred into embedding cassettes for lipid clearing. Passive lipid clearing was performed for 5 days at 40°C in the clearing solution [8% SDS (wt/vol), 0.2 M boric acid, pH adjusted to 8.5] under gentle agitation. Subsequently, the samples were thoroughly washed in PBSt for 2 days at room temperature with gentle agitation.

### Immunostaining of clarified samples

CLARITY-processed larvae were incubated in blocking solution [0.01 M PBS, 0.1% Tween 20 (vol/vol), 1% Triton X-100 (vol/vol), 10% DMSO (vol/vol), 10% normal goat serum (vol/vol), 0.05 M glycine] overnight at 4°C. Subsequently, samples were incubated in staining solution [0.01 M PBS, 0.1% Tween 20 (vol/vol), 0.1% Triton X-100 (vol/vol), 10% DMSO (vol/vol), 2% normal goat serum (vol/vol), 0.05% azide (vol/vol)] with primary antibodies [chicken polyclonal to GFP (Avès Labs, GFP-1010,1:600); rabbit polyclonal to DsRed (Clontech, 632496, 1:300)] for 5 days at room temperature under gentle agitation. After four washing steps in PBSt, samples were incubated in staining solution with secondary antibodies [Alexa Fluor 488-labelled goat anti-chicken (Invitrogen, A11039, 1:600); Alexa 555-labelled goat anti-rabbit (Invitrogen, A-21428, 1:300)] for 5 days at room temperature. Samples were washed for 2 days in PBSt and stained with 1 µM DiIC18(3) solution (DiI Stain; Molecular Probes).

### Imaging of clarified samples in high refractive index solution

A fructose-based high refractive index solution (fbHRI) was prepared as follows: 70% fructose (wt/vol), 20% DMSO (wt/vol) in 0.002 M PBS, 0.005% sodium azide (wt/vol). The refractive index of the solution was adjusted to 1.4571 using a refractometer (Kruss). In preparation for imaging, the samples were incubated in 50% (vol/vol) fbHRI for 6 h and finally incubated in fbHRI for at least 12 h. For imaging, samples were mounted in 1% (wt/vol) low-melting-point agarose and covered with fbHRI. Fluorescence of whole-mount larvae was recorded with a Leica TCS SP8 two-photon microscope equipped with a mode-locked Ti:Sapphire laser (Chameleon, Coherent) at 770 nm and the Leica HC FLUOTAR L 25×/1.00 IMM motCorr objective.

### Quantification of BBB leakage *in vivo*

The confocal images taken after IV dextran-FITC injection were analysed using ImageJ (http://imagej.nih.gov/ij/) as previously described (Watanabe et al., 2012). For each larva, two separate focal planes were selected. In each plane, five circular regions of interest (ROI) 15 μm in diameter were selected in the BP outside the CBV. Twenty single-pixel ROIs were selected in the CBV. The FI was calculated for each ROI (circular and single pixel) as well as the relative FI (BP/CBV).

### Quantification of GFP infection levels in eye-injected larvae

The confocal images taken after injection of SINV or CHIKV into the eye were analysed using ImageJ. For each larva, two ROI were selected: the eye, and the contralateral region of the optic tectum. A threshold was applied to each ROI, to remove background noise (e.g. autofluorescence coming from the pigments in the eye). Infection levels were calculated as the ratio between GFP in the optic tectum and the eye.

### Macrophage depletion

Metronidazole-mediated depletion was performed as described by Palha et al. (2013). Briefly, Tg*(mpeg:Gal4FF)^gl25^* fish (Ellett et al., 2011) were crossed with Tg*(UAS-E1b:Eco.NfsB-mCherry)^c264^* (Davison et al., 2007) to generate double-positive transgenics and single-positive sibling controls. Embryos were placed from 48 to 72 hpf in a 10 mM metronidazole, 0.1% DMSO solution to induce specific depletion of NfsB-mCherry-expressing macrophages. Embryos were then rinsed three times with embryo water and subsequently injected with the virus.

### Statistical analysis

To evaluate the difference between means, a two-tailed unpaired *t*-test or an analysis of variance (ANOVA) followed by Bonferroni's multiple comparison test were used, when appropriate. Normal distributions were analysed with the Kolmogorov–Smirnov test. Non-Gaussian data were analysed with a Kruskal–Wallis test followed by Dunn's multiple comparison test. A value of *P*<0.05 was considered statistically significant (***P<0.001; **P<0.01; *P<0.05; ns, not significant). Survival data were plotted using the Kaplan–Meier estimator, and log-rank tests were performed to assess differences between groups. Statistical analyses were performed using Prism 5 software (GraphPad).
